# Intrinsic Motivation and Sophisticated Epistemic Beliefs Are Promising Pathways to Science Achievement: Evidence From High Achieving Regions in the East and the West

**DOI:** 10.3389/fpsyg.2021.581193

**Published:** 2021-02-19

**Authors:** Ching Sing Chai, Pei-Yi Lin, Ronnel B. King, Morris Siu-Yung Jong

**Affiliations:** ^1^Department of Curriculum and Instruction, Centre for Learning Sciences & Technologies, The Chinese University of Hong Kong, Shatin, Hong Kong; ^2^Department of Education, National Kaohsiung Normal University, Kaohsiung, Taiwan; ^3^Faculty of Education, University of Macau, Macau, China

**Keywords:** intrinsic motivation, instrumental motivation, epistemic beliefs, science achievement, PISA 2015, Eastern and Western learners

## Abstract

Research on self-determination theory emphasizes the importance of the internalization of motivation as a crucial factor for determining the quality of motivation. Hence, intrinsic motivation is deemed as an important predictor of learning. Research on epistemic beliefs, on the other hand, focuses on the nature of knowledge, and learning with more sophisticated epistemic beliefs associated with more adaptive outcomes. While learning and achievement are multiply determined, a more comprehensive theoretical model that takes into account both motivational quality and epistemic beliefs is needed. Hence, this study aims to examine the role of intrinsic and instrumental motivation alongside epistemic beliefs in predicting students’ achievement in science. Data were drawn from the PISA 2015 survey. We focused on four of the top-performing societies. Two were Eastern societies – Singapore and Hong Kong, and the other two were Western societies: Canada and Finland. We found both common and specific patterns among the four societies. Regarding the common patterns, we found that intrinsic motivation and epistemic beliefs had direct positive effects on science achievement. As for the regionally-specific findings, instrumental motivation positively predicted achievement only in Western societies (i.e., Finland and Canada), but not in Eastern societies (i.e., Singapore and Hong Kong). The interaction effect between motivation and epistemic beliefs also demonstrated different patterns across the four societies. Implications for the role of motivation and epistemic beliefs in optimizing student learning and achievement are discussed.

## Introduction

Scientific and technological advances have greatly improved human life. In addition, emerging global issues such as the Covid-19 pandemic, global warming, and food shortage could only be resolved with more people having strong scientific knowledge and scientific ways of knowing. Despite the critical importance of science, not many students aspire to become scientists (Nugent et al., 2015). Moreover, there is a worrying trend that students’ intrinsic motivation to learn science in school and their aspiration to engage in a science-related career declines from elementary to high school (Alexander et al., 2012; Potvin and Hasni, 2014). Students may also possess unsophisticated assumptions about what science is and how it works (Li et al., 2018). Hence, there is a clear need to look into students’ motivation and science-related epistemic beliefs.

Identifying the factors that would optimize science learning and achievement is an urgent educational issue. In this study, we focus particularly on the role of motivation and epistemic beliefs in predicting science achievement. This study is novel because it integrates research on motivation which usually focuses on *why* students learn science with research on epistemic beliefs which pertains to students’ perceptions of *what* science is. While these two bodies of research have been quite active (e.g., Chen et al., 2014; Lin and Tsai, 2017), there is little research attempt to study them together. There is theoretical value in exploring their synergies as science learning is likely to be multiply determined. Researchers have increasingly warned against devoting exclusive attention to one key variable and neglecting a broader view of the critical factors underpinning key outcomes (Pettigrew and Hewstone, 2017; Yarkoni and Westfall, 2017).

Students who have high levels of motivation have a “why” for engaging in science-related learning activities. These students might be either intrinsically motivated as they just love learning science for its own sake or instrumentally motivated when they engage in learning science to advance their careers or to graduate from school. However, being motivated might not be enough to yield high levels of achievement. Numerous studies have shown that the relationship between motivational factors and achievement though statistically significant is smaller than other psycho-educational factors (e.g., Hulleman et al., 2010; Howard et al., 2017; Kriegbaum et al., 2018). This suggests the need to examine other potentially important factors that underpin science achievement and learning.

This brings us to the importance of recognizing that optimal science learning happens when students have a strong *why* (i.e., motivation) but also have a sophisticated understanding *what* science and scientific knowledge is all about. The investigation of epistemic beliefs about scientific knowledge is increasingly important in a post-truth society where scientific truth is contested and when an increasing number of people hold unscientific beliefs (e.g., Hornsey and Fielding, 2017; Hornsey et al., 2018). For example, researchers have found a large number of individuals holding anti-vaccination beliefs and harboring skepticism about climate change (Ecklund et al., 2017; Rizeq et al., 2020). These trends are associated with a strong resistance to evidence-based reasoning posing serious threats to societal progress (Hornsey and Fielding, 2017). Research on epistemic beliefs may hold potential implications for these critical problems (Hartman et al., 2017; Wilson, 2018).

Hence, the main research objective of this study is to explore the role of both motivation and epistemic beliefs in predicting science learning. To achieve this objective, we analyze data from the Program for International Student Assessment (PISA) from four different regions (Singapore, Hong Kong, Finland, and Canada) representing high-achieving societies across both East and West thereby allowing us to identify the possible cross-cultural factors that are common in predicting science achievement.

This study also addresses methodological shortcomings of past research. Past studies on science learning and achievement have been hampered by their exclusive focus on one cultural context (Chen et al., 2014; Lin and Tsai, 2017; Wong et al., 2019; Kaderavek et al., 2020). Hence, the possible cross-cultural applicability of the results might be questioned. This is a particularly important issue as researchers have shown the importance of culture in influencing students’ learning and motivational processes and their epistemic beliefs (Zusho and Clayton, 2011; Lee et al., 2012; King and McInerney, 2014; King et al., 2018).

In an attempt to address how epistemic beliefs may influence academic achievement, Greene et al.’s (2018) meta-analysis revealed that sophisticated epistemic beliefs (i.e., adaptive view on the development and justification of knowledge as constructed and evidence-based) are more influential of academic achievement than unsophisticated epistemic beliefs (i.e., view knowledge as absolute and certain). In addition, most epistemic beliefs studies have primarily relied on self-reports (see Debacker et al., 2008), and its effect on achievement needs to be further explored. It thus becomes important to identify the generalizations and contextually adaptive views on knowledge and knowing when assessing what constitutes a set of sophisticated beliefs in a certain discipline.

Hence, the purpose of this study was to empirically examine an integrated theoretical framework to assess whether students’ motivations, epistemic beliefs, and the interaction between their motivation and epistemic beliefs are predictive of science achievement across different societies representing different cultures.

## Literature Review

### Motivation to Learn Science

Student motivation to refers to why students undertake a learning task (Deci and Ryan, 2000; Pintrich, 2003). Though motivation is a complex phenomenon, self-determination theory suggests a common model that explains the process of how learners’ innate behavior and inherent propensity drive them to accomplish the desired educational outcomes (Elliott and Dweck, 1988; Deci et al., 1991; Deci and Ryan, 2000). Students who are intrinsically motivated view learning science as interesting and working on scientific issues enjoyable (Ryan and Deci, 2009). Studies have shown that students who are intrinsically motivated in science participate more in science-related activities (Lin and Schunn, 2016), and these factors would consequently influence students’ science achievement (Burns et al., 2019).

On the other hand, instrumental motivation (also called utility value) to learn science reflects students’ desire to learn science as a means to achieve a certain goal (i.e., to pursue further studies or for career progression) (Nagengast and Marsh, 2014). Instrumental motivation is a predictor of achievement and career choice (Canning et al., 2018). Previous research supports that students were more likely to learn science when they perceived the instrumental value of studying science in order to attain STEM-related career expectations or have successful work outcomes later on (Rozek et al., 2015). Nonetheless, instrumental motivation seems to have weaker association with science achievement compared to intrinsic motivation (Liang and Tsai, 2010).

More importantly, the two types of motivations could co-exist; an individual can be both instrumentally and intrinsically motivated (Hidi and Harackiewicz, 2000). In this study, we investigated motivational variables (i.e., intrinsic motivation and instrumental motivation) in predicting science achievement across the selected societies.

### Epistemic Beliefs About Science

Epistemology is a sub-discipline of philosophy that is concerned with the nature and grounds of knowledge, and ways of knowing (Hofer, 2002). Within the fields of psychology and education, epistemic beliefs focus on students’ beliefs about the nature of knowledge and knowing process (Schommer, 1990; Hofer and Pintrich, 1997; Hofer, 2002). The evolution of the thinking process about knowledge and knowing has become prominent in science education (Scott et al., 2006; Lin and Tsai, 2017). In general, science epistemic beliefs are associated with students’ scientific reasoning, interpreting and justifying scientific ideas based upon empirical evidence and through critical thinking (Hofer and Pintrich, 1997).

In this study, epistemic beliefs are posited as students’ beliefs about science and scientific knowledge. This involves how students scientifically explain phenomenon, interpret data and evidence, and approach science issues (OECD, 2016a). Students with sophisticated epistemic beliefs are more likely to hold intrinsic goal orientation to make inferences and comparisons from one or multiple texts, construct perspectives from integrated information, and apply scientific ideas and concepts to make evaluations and justifications (Paulsen and Feldman, 2005; Chen and Pajares, 2010; Tsai et al., 2011). Most importantly, sophisticated epistemic belief entails an understanding about the evolving and constructed nature of scientific knowledge (Muis, 2007; Krist, 2020).

Sophisticated epistemic beliefs about science generally are associated with higher levels of achievement (Greene et al., 2018). In addition, middle-school students with sophisticated epistemic beliefs undertake scientific inquiry in a qualitatively different manner. They could use scientific standards to provide insights into their understanding of the explanatory and descriptive goals, conceptual coherence and clarity, and empirically evidence evaluation for scientific models (Pluta et al., 2011; Belland et al., 2016).

In the domain of science, PISA measures students’ sophisticated epistemic beliefs about science as tentative and evolving. Epistemic beliefs encompass students’ views about the need for scientific experiments to justify scientific knowledge, and a recognition of the limitations of scientific experiments (OECD, 2016a). The investigation of epistemic beliefs about science is extremely important in the context of a post-truth society where it is imperative that students develop the skills to evaluate scientific evidence and explanations (Sinatra and Lombardi, 2020).

### Relationship Between Motivations and Epistemic Beliefs

Past research has explored the associations among learning motivation, epistemic beliefs and achievement, and indicated that students’ motivation and how they view science impact the learning process (e.g., Chen, 2012; Mason et al., 2013; Ho and Liang, 2015). Research has indicated that students with a strong intrinsic motivation tend to invest their time and effort in seeking in-depth understanding (Chen and Pajares, 2010; Burns et al., 2019). For example, students’ intrinsic motivation is associated with adopting constructive learning strategies to construct scientific knowledge (Lin et al., 2013; Ho and Liang, 2015; Shen et al., 2018). Nonetheless, the decline of students’ motivation to learn science (Vedder-Weiss and Fortus, 2011) and promotion of students’ sophisticated epistemic beliefs (Lee et al., 2016) are critical issues. Therefore, this study aimed to explore the generalizability of motivation, epistemic beliefs, and achievement across societies.

### Commonality and Specificity

A critical issue in examining the pattern of relationships among the variables is whether they are common across cultures or whether they are culturally-specific. Much of the existing research in motivation and epistemic beliefs have been conducted in WEIRD (Western, educated, industrialized, rich democratic societies) (Henrich et al., 2010). Though many motivational phenomena are commonly observed across different cultures (e.g., Pintrich, 2003), students may also have different motivational orientations (e.g., Brown et al., 2018; Liu et al., 2020). The critical factors that underpin learning and achievement are also strongly influenced by sociocultural factors (Chiu and Chow, 2010; Chiu et al., 2016; King and McInerney, 2019; Li and Yamamoto, 2020). Hence, it is important to test the cross-cultural applicability of the models (King and McInerney, 2014; King et al., 2018).

Science epistemic beliefs, which refer to individuals’ beliefs about the nature of knowledge and knowing has been found to be associated with cultural factors (Hofer, 2008). For example, Schommer-Aikins and Easter (2008) argued that Euro-American students had significantly higher epistemic belief scores (i.e., student beliefs about the speed of knowledge acquisition and knowledge construction and modification) compared to Asian American students. More recently, Yang (2016) reviewed 106 studies and concluded that there are cultural differences with epistemic beliefs in the context of science learning. More specifically, it seems that American and Taiwanese students may have more sophisticated epistemic beliefs, while Turkish and Chinese students may rely more on authority.

Cultural differences are also reflected in teaching practices. In Asian educational contexts, science learning is dominated by traditional didactic approaches wherein students are asked to provide certain and correct answers (e.g., Ho and Liang, 2015). In contrast, science learning in Western societies is more dominated by inquiry-based approaches which could foster more sophisticated epistemic beliefs (Yang, 2016). Hence, further comparative work is needed to explore the contextually and culturally situated nature of epistemic beliefs.

## Science Learning Context in Singapore, Hong Kong, Finland and Canada

Given the excellent performance by Singapore, Finland, Canada, and Hong Kong in the science literacy test in PISA 2015, an introduction to the four societies’ science learning context will allow for better interpretations of students’ learning motivation, science epistemic beliefs, and its relation to science literacy. We focus on these four societies given that they represent high-performing regions in the West and the East. Moreover, all four societies are considered highly economically developed thereby minimizing potential confounds.

We are aware that these four societies do not completely represent the West and the East as there are numerous countries that could be classified into West–East. Hence, we invite readers to be cautious in making over-generalizations. Adding too many societies, however, would work against model parsimony as there might be too many country-level confounds that might potentially bias the results (e.g., differences in geography, cultural values, climate, political system, demographic profile) (e.g., Oishi, 2014; Krems et al., 2017). For example, though Estonia is also a top-performing Western country, the country’s governance and cultural values differ from Canada and Finland. Similarly, one could classify Vietnam as a top-performing Eastern country but it is demographically very different from Hong Kong and Singapore which both share a British colonial history and have relatively similar economic profiles. Bearing this caveat in mind, we discuss each of the four societies we included in our study.

### Singaporean Context

In the Singapore education system, science classes start in the 3rd grade and in secondary schools, students will learn general science until the eighth grade. The center of science education is focused on promoting “science as an inquiry” for students to relate science to society, daily life, and the environment (Ministry of Education [MOE], 2013, 2014). The curriculum emphasizes students’ acquisition of science knowledge, understanding, and application; scientific skills and knowing processes; and scientific attitudes with ethical handling of scientific issues (Ministry of Education [MOE], 2013, 2014). Recent studies indicate that the inquiry-oriented science pedagogy enhances Singaporean students’ interest in school science and science learning (Jocz et al., 2014; Sun et al., 2016). Nonetheless, only a small group of students in Singapore reported that they like learning science in TIMSS 2011 assessment, and thus examination of students’ science motivation is considered in the current study (Lay and Chandrasegaran, 2016).

### Hong Kong Context

The science education system in Hong Kong is implemented in the general studies curriculum at the primary school level that integrates the disciplines of social science, science, and technology, and at the secondary school level, science education is positioned to strengthen students’ science knowledge and ability to integrate and apply science knowledge across disciplines (Curriculum Development Council, 2017). Science inquiry is positioned as a pedagogical means to engage students in acquiring science knowledge and advanced scientific skills (Wan and Lee, 2017; Cheung, 2018), and to prepare students’ readiness for the workplace and solving daily life problems (Jong, 2017; So et al., 2018). Moreover, the national science curriculum guidelines also highlights the importance of enhancing students’ motivation through connecting science-related issues to their daily life, and encourages teachers to adopt inquiry-based, or hands-on activities to develop students’ interest in science (Curriculum Development Council, 2017).

### Finnish Context

In Finland, primary science education (Grades 1–6) is taught as an integrated course that aims to transmit the nature of science (Finnish National Agency for Education, 2017). At the secondary school level, science could be taught as an integrated subject or as more specialized into the separate subjects of physics, chemistry, geography, and biology (Lavonen and Juuti, 2016). The Finnish science curriculum may be characterized as an inquiry- or context-based approach to raise students’ interest and motivation toward science subjects (Kang et al., 2019; Lehtinen et al., 2019). It highlights the importance of personal relevance by linking science content to their lives, which apparently leads to a positive correlation with interest and achievement (Kang and Keinonen, 2018). Past research showed that, compared to students in the United States, Finnish students felt confident, successful, and happy during their science classes (Schneider et al., 2016).

### Canadian Context

In Canada, science education varies across the 13 jurisdictions (Milford and Tippett, 2019). The Council of Ministers of Education, Canada (2016) aims that students develop (i) an understanding of the nature of science, technology, society, and the environment (STSE), (ii) scientific and technological inquiry, (iii) knowledge in life sciences, physical sciences, and earth and space sciences, and (iv) attitudes that support the scientific and technological acquisition and application. Studies have shown that Canadian students are able to extend and deepen their understanding of fundamental science concepts and learn to use science knowledge and processes as a scientist does (Hasni et al., 2016; Asghar et al., 2019). On the other hand, in a local study conducted by Potvin and Hasni (2014), there is a slight decrease of students’ interests in science learning from 5-grade through 11-grade.

The present study includes data from the four top-performing countries and regions, and aims to investigate whether there is a general relationship with the four factors – science epistemic beliefs, intrinsic motivation, instrumental motivation, and science achievement – assessed in PISA 2015. The research questions are:

1. Do students’ science motivations (i.e., intrinsic and instrumental motivation) predict their science achievement?
2. Do students’ epistemic beliefs predict their science achievement?
3. Do students’ motivations, epistemic beliefs, and the interaction between motivations and epistemic beliefs predict their science achievement?

## Methods

### Sample

The sample for this study adopted data released from the PISA 2015 database. PISA 2015 measured how 15-year-old students in 72 participating countries and regions meet the challenges of today’s knowledge societies (OECD, 2016a). In 2015, science was the major assessment domain. The present study includes two Eastern societies – Singapore, Hong Kong – and two Western societies – Finland and Canada – from the top-10 performing countries and regions in PISA 2015 to validate a cross-contexts comparison. The total number of participants from all participating countries and regions was 418,458 students (50.1% female). In this study, we only focused on four societies: the Singapore 5,748 students (48.6% female); Hong Kong 5,011 students (49.9% female), Canada 17,220 students (50% female), and Finland 5,060 students (48.7% female).

### Variables

The Program for International Student Assessment is an international assessment administered by the OECD. PISA data were examined in different analyses to ensure the quality of data meet designed criteria. Research also has used PISA 2015 to provide insight into students’ science learning and literacy (Aditomo and Klieme, 2020; Tang and Zhang, 2020). In the current study, variables were chosen from the student questionnaire in PISA 2015. This study includes the following variables taken from the student questionnaire in PISA 2015.

#### Intrinsic Motivation to Learn Science

Intrinsic motivation pertains to students’ enjoyment of engaging in science learning activities based on their responses to questions such as whether they have fun when learning science topics, like reading about science, enjoy learning new science topics and acquiring new knowledge in science. PISA 2015 measures students’ enjoyment of learning science through a four-point Likert scale from “1 = *strongly disagree*” to “4 = *strongly agree*.” A sample item is, “I have fun when I am learning <broad science>.” Higher levels agreement indicates that students enjoy learning science for its own sake. Reliabilities (Cronbach’s α) measured in this study ranged from 0.93 to 0.96, which was in line with OECD’s technical report (2016b).

#### Instrumental Motivation to Learn Science

Instrumental motivation measured students’ agreement to whether that making an effort to learn science is worthwhile because school science is helpful for later-on work and career plans. Students’ responses on a four-point Likert scale with categories from “1 = *strongly agree*” to “4 = *strongly disagree*.” The responses were reverse-coded so that higher values refer to higher levels of instrumental motivation. A sample item is, “Studying my <school science> subject(s) is worthwhile for me because what I learn will improve my career prospects.” Reliabilities (Cronbach’s α) measured in this study ranged from 0.91 to 0.95, which was in line with OECD’s technical report (2016b).

#### Epistemic Beliefs About Science

Epistemic beliefs about science investigated students’ views on scientific approaches, understanding of scientific knowledge as derived from experimentation, and that scientific knowledge is revisable based on the experimental evidence. A four-point Likert scale with the answering categories from “1 = *strongly disagree*” to “4 = *strongly agree*” was measured. A sample item is, “Good answers are based on evidence from many different experiments.” Higher levels of agreement indicate that students possess more sophisticated epistemic beliefs about science. Reliabilities (Cronbach’s α) measured in this study ranged from 0.88 to 0.91, which was in line with OECD’s technical report (2016b).

#### Science Achievement

The PISA 2015 science achievement score was viewed as the cognitive learning outcome in this study. The PISA 2015 described a clear framework in measuring students’ scientific competencies (i.e., explain phenomena scientifically, evaluate and design scientific inquiry, and interpret data and evidence scientifically). The test content is not confined by school science content, but rather by contexts and problems for which science knowledge, scientific methods can be applied.

### Data Analyses

Data were analyzed in accordance with the research questions of the study. Firstly, the univariate normality was examined in accordance with Kline’s (2005) criteria. The values of skewness (ranged from −1.02 to −0.79) and kurtosis (ranged from −0.70 to 0.10) (see Table 1) indicated the dataset was normally distributed following the recommended value that skewness and kurtosis should be under | 3| and | 10|, respectively. In the preliminary analyses, exploratory factor analyses (EFA) with SPSS version 21 (IBM Corp. Released 2012. IBM SPSS Statistics for Windows, Version 21.0. Armonk, NY, United States: IBM Corp.) were employed to examine the construct validity of the responses to the Singapore, Hong Kong, Finland, and Canada datasets. A-three factor (i.e., intrinsic motivation, instrumental motivation, and epistemic beliefs) model was established.

**TABLE 1:** Means and SD of measured items.

	All PISA participants (*N* = 418458)		Singapore (*n* = 5748)		Hong Kong (*n* = 5011)		Canada (*n* = 17220)		Finland (*n* = 5060)	
	Mean	SD	Mean	SD	Mean	SD	Mean	SD	Mean	SD
1. Intrinsic motivation	2.73	0.78	3.01	0.68	2.80	0.75	2.85	0.80	2.57	0.73
2. Instrumental motivation	2.90	0.79	3.08	0.65	2.85	0.77	3.04	0.78	2.79	0.75
3. Epistemic beliefs	3.02	0.58	3.15	0.50	3.06	0.54	3.18	0.59	2.99	0.56
Skewness	−0.90	−0.25	−0.94	−0.51	−0.79	−0.28	−1.02	−0.26	−0.84	−0.03
Kurtosis	−0.19	1.04	0.10	2.08	−0.47	2.25	−0.70	1.65	−0.55	1.91

Pearson’s correlation analysis was conducted. In PISA 2015, there were 10 plausible values that presented students’ achievement, we conducted plausible values analysis using each plausible value separately, then, computed and averaged them (OECD, 2009). Multilevel modeling is used to analyze data because students were nested in schools. This study employed a two level model (level 1 = student level, level 2 = school level) to examine the influence of schools on students’ science achievement. We ran four multilevel models for each region. The first model was a null model to partition the between- and within-groups variance in science achievement. The intra-class correlation coefficient (ICC) is the ratio of between-group variance to the total variance. In the second model, we specified a random intercept model. The following level 1 predictors were included: gender, students’ economic, social, and cultural status (which is based on students’ scores in PISA 2015 ESCS measure) intrinsic motivation, and instrumental motivation. The third model is also a random intercept model that included the following predictors: gender, ESCS and epistemic beliefs. The fourth model is a full model including interaction effects. The predictors were: gender, ESCS, intrinsic motivation, instrumental motivation, epistemic beliefs, and interaction between motivation and epistemic beliefs. Gender and ESCS measure are controlled as covariates to predict science achievement in the model 2 to 4. The data file downloaded from OECD website^1^ is weighted at the student level with normalized student final weights (OECD’s technical report, 2016b) and listwise deletion is used to treat missing data.

## Results

The results are presented in the following sections. First, preliminary analyses included the EFA and the bivariate correlations, established a structural model and explored relationships between students’ intrinsic motivation, instrumental motivation, and epistemic beliefs among the four countries and regions. The main analyses were about examining how intrinsic motivation and instrumental motivation, and epistemic beliefs and their interactions predict science achievement.

### Establishing the Factor Structure

We first tested the factor structure using exploratory factor analysis to examine the factors of the measurement. Principal axis factor analyses with direct oblimin rotation were run on the data. A three-component structure among the four selected societies was established. Follow Hair et al.’s (2010) recommendation, three latent factors were specified by factor loadings greater than 0.5, and eigenvalues greater than one. The intrinsic motivation includes five items, the instrumental motivation includes four items, and the epistemic beliefs includes six items, of the measurement are listed in Appendix 1.

The Kaiser–Meyer–Olkin (KMO) Value and Bartlett’s test of sphericity were calculated before the EFA to determine the applicability of the factor analyses. In the present study, all KMO values greater than 0.50 (KMO = 0.91 in the Singapore, Canada, and Finland dataset; KMO = 0.93 in the Hong Kong dataset; see Table 2) indicated that factor analysis sampling was appropriate. Bartlett’s test of sphericity indicated significance for EFA (X^2^ = 67809.839, df = 105, *p* < 0.001 in the Singapore dataset; X^2^ = 72282.874, df = 105, *p* < 0.001 in the Hong Kong dataset; X^2^ = 219084.577, df = 105, *p* < 0.001 in the Canada dataset; X^2^ = 61184.599, df = 105, *p* < 0.001 in the Finland dataset; see Table 2). Factor loadings of measured items ranged from 0.68 to 0.94 in the Singapore dataset; ranged from 0.70 to 0.94 in the Hong Kong dataset; ranged from 0.72 to 0.93 in the Canada dataset; and ranged from 0.72 to 0.91 in the Finland dataset (see Table 2). Total explained variance was found to be 68.82% in the Singapore dataset; 75.80% in the Hong Kong dataset; 73.05% in the Canada dataset; and 71.23% in the Finland dataset.

**TABLE 2:** EFA of measured items.

	Singapore		Hong Kong		Canada		Finland	
	Factor loadings	% of variance	Factor loadings	% of variance	Factor loadings	% of variance	Factor loadings	% of variance
1. Intrinsic motivation	0.87–0.94	40.20	0.80–0.94	46.67	0.85–0.92	40.55	0.81–0.91	38.98
2. Instrumental motivation	0.77–0.90	12.56	0.88–0.93	10.94	0.84–0.93	12.48	0.86–0.91	12.48
3. Epistemic beliefs	0.68–0.77	16.07	0.70–0.86	18.20	0.72–0.83	20.03	0.72–0.82	19.77
Kaiser-Meyer-Olkin value	0.91		0.93		0.91		0.91	
Bartlett’s test of	X^2^ = 67809.839 df = 105		X^2^ = 72282.874 df = 105		X^2^ = 219084.577 df = 105		X^2^ = 61184.599 df = 105	
sphericity	*p* < 0.001		*p* < 0.001		*p* < 0.001		*p* < 0.001	
Total % of variance	68.82		75.80		73.05		71.23	

Next, we addressed the relationships among the latent factors in Table 3. Correlations were computed for Singaporean, Hong Kong’s, Canadian, and Finnish students. Intrinsic motivation, instrumental motivation and epistemic beliefs were all positively and significantly correlated (ranging from 0.09 to 0.50). The lowest correlation was found for the association between instrumental motivation and science achievement in the Singapore dataset. High correlations between intrinsic motivation and instrumental motivation were found in the four societies. Regarding science achievement, epistemic beliefs were strongly and positively correlated to science achievement in the Hong Kong and Finland datasets, whereas intrinsic motivation was found to be strongly and positively related to science achievement in the Singapore and Canada datasets.

**TABLE 3:** Correlations of motivations, epistemic beliefs, and science achievement.

	1	2	3	4	1	2	3	4
1. Intrinsic motivation	–	0.50	0.41	0.26	–	0.41	0.29	0.32
2. Instrumental motivation	0.39	–	0.26	0.12	0.41	–	0.15	0.18
3. Epistemic beliefs	0.37	0.21	–	0.28	0.32	0.16	–	0.37
4. Achievement	0.33	0.09	0.29	–	0.33	0.16	0.31	–

*All correlations were statistically significant at p < 0.001. The lower triangle in the left column is the Singapore data; the upper triangle in the left column is the Hong Kong data. The lower triangle in the right column is the Canada data; the upper triangle in the right column is the Finland data.*

### Predicting Students’ Science Achievement

We hypothesized that (i) motivations (i.e., intrinsic and instrumental motivation), (ii) epistemic beliefs, and (iii) their interactions would predict science achievement when entered separately into the regression equation. We analyzed the predictive effect of science achievement using four models to respectively, answer our three research questions in Table 4. The ICC for model 1 was 35% in Singapore, 32% in Hong Kong, 16% in Canada, and 9% in Finland. The intercepts varied significantly across schools (Wald *Z* = 8.53, *p* < 0.001, in Singapore; Wald *Z* = 7.83, *p* < 0.001, in Hong Kong; Wald *Z* = 15.45, *p* < 0.001, in Canada; Wald *Z* = 6.44, *p* < 0.001, in Finland). The results support the use of multilevel modeling.

**TABLE 4:** Multilevel analyses for predicting science achievement.

	Singapore				Hong Kong			
	Model 1	Model 2	Model 3	Model 4	Model 1	Model 2	Model 3	Model 4
Intercept	545.07***	456.69***	427.90***	363.77***	524.81***	461.57***	433.44***	422.87***
*Level 1*								
Gender (female)		–4.25	−7.82**	–4.02		−7.18***	−11.82**	−8.00***
ESCS (SES)		24.17***	25.47***	23.23***		3.22**	3.63**	2.58*
INTR		35.61***		20.05**		25.48***		12.73*
INST		−4.48**		12.19*		–0.51		–0.35
EB			39.19***	37.65***			32.89***	19.38***
INTR × EB				3.20				2.17
INST × EB				−6.03**				–0.74
Residual variance	7151.25	6133.35	6282.84	5953.52	4398.33	3982.80	4015.12	3849.49
Intercept variance (School level)	3838.69	2317.38	2373.90	2184.47	2027.22	1881.81	1779.16	1800.18
Intra-class correlation	0.35	0.27	0.27	0.27	0.32	0.32	0.31	0.31
Model fit: −2 Log likelihood	72142.68	68465.16	68308.00	66607.25	60563.80	56819.86	57234.82	55655.61

	**Canada**				**Finland**			
	**Model 1**	**Model 2**	**Model 3**	**Model 4**	**Model 1**	**Model 2**	**Model 3**	**Model 4**

Intercept	514.37***	413.86***	384.11***	329.27***	530.58***	426.52***	374.60***	373.44***
*Level 1*								
Gender (female)		−2.75*	−6.01***	−4.29**		14.79***	10.52***	11.41***
ESCS (SES)		20.63***	22.17***	19.20***		30.24***	29.78***	26.10***
INTR		31.43***		18.60***		33.59***		12.72*
INST		2.63**		12.04**		3.58*		–8.96
EB			40.39***	34.04***			50.39***	19.98**
INTR × EB				2.03*				4.31
INST × EB				−3.23**				3.91*
Residual variance	7086.60	5970.55	6113.90	5703.93	8384.53	6827.76	6663.75	6251.62
Intercept variance (School level)	1361.85	826.82	736.05	722.14	797.44	355.39	275.04	266.65
Intra-class correlation	0.16	0.12	0.11	0.11	0.09	0.05	0.04	0.04
Model fit: −2 Log likelihood	236063.32	207675.21	208073.18	198240.75	70065.23	62252.85	61088.81	58630.71

INTR, intrinsic motivation; INST, instrumental motivation; EB, epistemic belief; Gender: Control variable (1 = female, 2 = male). All the residual variance and intercept variance are significant at ***p < 0.001 level. *p < 0.05; **p < 0.01; ***p < 0.001.

In model 2, gender and ESCS were control covariates. Intrinsic motivation and instrumental motivation were entered as independent variables. The results indicated that intrinsic motivation significantly predicted science achievement across the four societies. Instrumental motivation was a negative predictor of science achievement in Singapore, yet, a positive predictor of science achievement in Canada and Finland.

In model 3, we found that epistemic beliefs were a positive predictor of science achievement across the four societies.

In model 4, intrinsic motivation and instrumental motivation, epistemic beliefs, and interaction of motivations and epistemic beliefs (i.e., intrinsic motivation × epistemic beliefs and instrumental motivation × epistemic beliefs) were entered as predictors of science achievement. In this model, intrinsic motivation and epistemic beliefs were both positively associated with science achievement across the four societies.

Gender differences across cultures were also observed. Males had higher science scores in Hong Kong and Canada, females scored higher than males in Finland. In Singapore, there was no gender difference. ESCS was a positive predictor in the four regions.

However, cross-cultural differences were observed as regards instrumental motivation. Instrumental motivation positively predicted science achievement only in the Western countries such as Canada and Finland. In the East (Singapore), instrumental motivation was a negative predictor. Instrumental motivation was not significantly related to achievement in Hong Kong.

**FIGURE 1:**
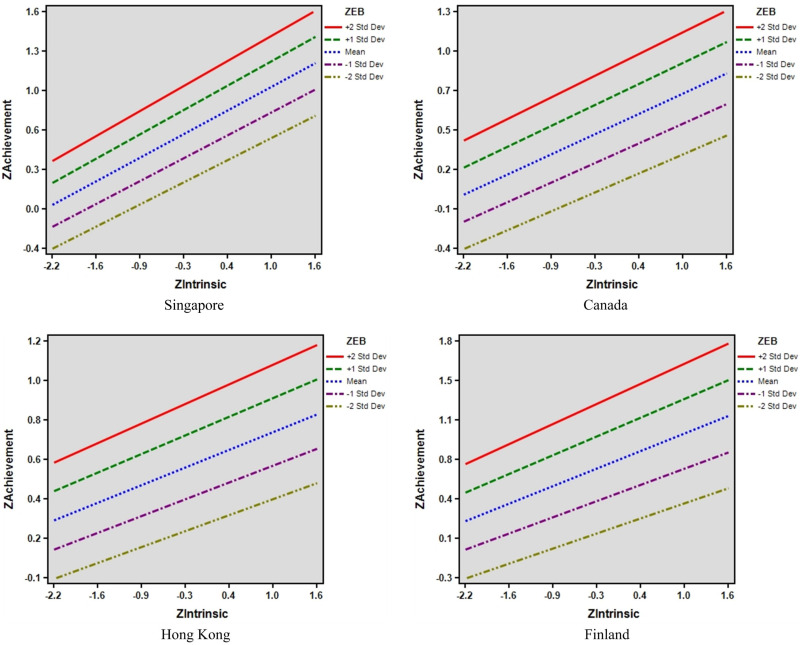
Predicting science achievement: a graphical illustration of interaction of intrinsic motivation × epistemic beliefs.

**FIGURE 2:**
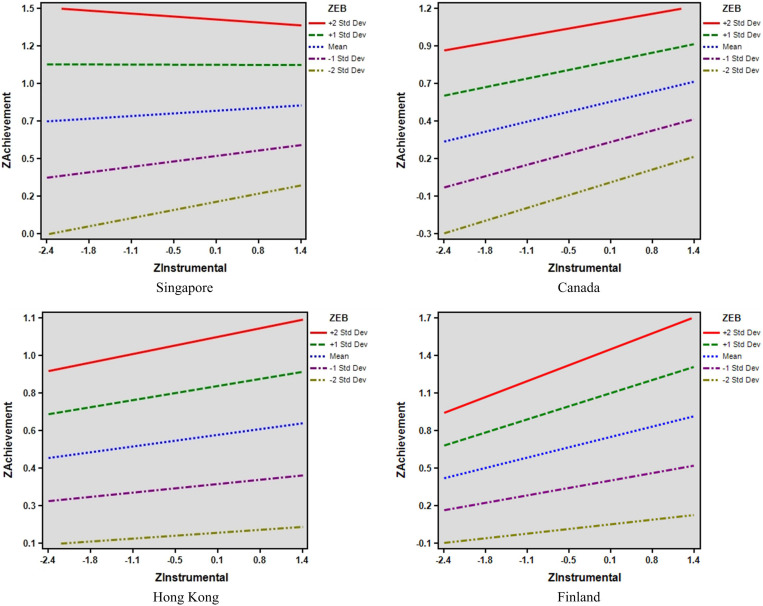
Predicting science achievement: a graphical illustration of interaction of instrumental motivation × epistemic beliefs.

To help with the interpretation of the finding, Figure 1 illustrates the interaction between intrinsic motivation and epistemic beliefs, while Figure 2 depicts the interaction between instrumental motivation and epistemic beliefs. The *X*-axis represents motivation (intrinsic or instrumental), while the *Y*-axis represents achievement. Science achievement was particularly high when both intrinsic motivation and epistemic beliefs were high in the four societies. This demonstrates that the factors are important across the four regions.

We also found culturally specific findings. In Singapore, students’ epistemic beliefs had a stronger association with achievement when instrumental motivation was low. In Singapore and Canada, students’ instrumental motivation had a stronger association with achievement when they had less sophisticated epistemic beliefs (e.g., −1SD and −2SD below the mean). In Finland, students’ instrumental motivation had a stronger association with achievement when they had sophisticated epistemic beliefs (e.g., +1SD and +2SD below the mean).

## Discussion

We examined the associations among intrinsic motivation, instrumental motivation, epistemic beliefs, and their interactions to predict science achievement in a large sample of 15-years old students across four societies. Given that studies about the interrelationships these factors are usually culturally specific (Chen et al., 2014; Lin and Tsai, 2017; Wong et al., 2019; Kaderavek et al., 2020), this study first established the construct validity of the factors measured for the four different societies. This effort allows us to discuss the findings with some confidence about cross-cultural applicability.

In relation to the first research question, we found empirical support that intrinsic motivation is predictive of students’ science achievement for the four regions. This finding extends the current understanding that intrinsic motivation could be a common factor that predicts science learning achievement (Ryan and Deci, 2009; Lin and Schunn, 2016; Burns et al., 2019). A practical implication of this finding is that teachers are encouraged to foster students’ intrinsic motivation to learn science regardless of cultural or contextual differences.

As for instrumental motivation, our findings indicate that it was positively associated with achievement in Canada and Finland, yet negatively associated with achievement in Singapore. The case of Canada and Finland may reflect a stronger emphasis in Western societies about the use of instrumental motivation to encourage students to learn science (Rozek et al., 2015; Canning et al., 2018). In the Asian context, Liang and Tsai (2010) reported a weaker association between instrumental motivation and achievement. Our finding also indicates that instrumental motivation is not a significant predictor for Hong Kong students’ achievement when both forms of motivation are considered. However, in model 4, instrumental motivation is a negative predictor for the Hong Kong sample. There could be a higher emphasis on the instrumental value of science in Hong Kong (So et al., 2018). In general, it seems that leveraging on instrumental motivation may not enhance students’ achievement in the two Eastern regions. In addition, given that the correlation between the achievement and the instrumental motivation is significant and positive (*r* = 0.09 for Singapore), the negative regression weight for the Singapore sample could be due to suppression effects. The situation warrants more specific cross-cultural research in this area.

Second, epistemic beliefs significantly predicted science achievement across all societies for both model 3 and model 4. The importance of facilitating development of sophisticated epistemic beliefs for science has received constant attention (Scott et al., 2006; Lin and Tsai, 2017). This study affirms the importance of epistemic beliefs for science achievement and science learning (Pluta et al., 2011; Belland et al., 2016; Greene et al., 2018) through the regression analyses among the four top-performing countries or regions. The implication to science education would be that epistemic beliefs about science need to be emphasized and explicitly discussed in class. The four samples we analyzed have a common emphasis on teaching science through inquiry with the aim of providing students with opportunities to be scientists rather than just science learners (Jocz et al., 2014; Cheung, 2018; Asghar et al., 2019; Inkinen et al., 2020). In particular, to develop more sophisticated epistemic beliefs, students need to be able to question knowledge claims and make justification from multiple references and sources (Belland et al., 2016).

Third, in model 4, intrinsic motivation and epistemic beliefs are both positive predictors of science achievement when both are entered into the regression equation (i.e., model 4), and this finding is commonly reflected across the four societies. This finding affirms previous research that has investigated the structural relationships among epistemic beliefs and motivation (Chen, 2012; Ho and Liang, 2015). In particular, Ho and Liang (2015) illustrate that sophisticated epistemic beliefs are predictive of deep intrinsic motivation to learn science mediated by constructive conceptions of learning science. This study extends the previous study with the science achievement as the predicted outcome to provide more support for science educators to structure intrinsically motivating science learning activities that concurrently challenges students to draw on sophisticated epistemic beliefs (Mason et al., 2013). Our finding also reveals that the interaction between intrinsic motivation and epistemic beliefs positively predicted science achievement in the Singapore dataset.

Instrumental motivation, on the other hand, showed a culturally specific pattern. In the Singapore context, instrumental motivation was a negative predictor of science achievement after taking into account the variance predicted by intrinsic motivation, whereas in the Western context, it is a positive predictor.

The interaction between instrumental motivation and epistemic beliefs showed culturally specific patterns. In Finland, the relationship between instrumental motivation and achievement was strongest for those with the most sophisticated epistemic beliefs. However, in Singapore and Canada, the relationship between instrumental motivation and achievement was strongest for students with less sophisticated epistemic beliefs. For those with more sophisticated epistemic beliefs, the relationship between instrumental motivation and achievement was weaker. These differential patterns might reflect differences in the educational system across countries though further research is needed to understand these patterns.

In this study, we also detected that gender influences achievement differently based on societies. Students’ SES was an influential predictive of science achievement. Science educators may therefore need to pay specific attention to the gender issue while they design interesting and enjoyable science learning activities, depending on where they are located. Overall, there was no gender difference in Singaporean students’ motivation and epistemic beliefs. In Hong Kong, male students had higher intrinsic motivation than female students in learning science. For Canadian students, male students had higher scores in motivation to learn science and epistemic beliefs than female students. On the other hand, Finnish female students had higher scores in motivation to learn science and epistemic beliefs than male students.

The results of the present study provide support for the complexity of factors that predict science achievement. We found that intrinsic motivation and epistemic beliefs are closely associated with science achievement, which may provide insights on the importance of intrinsic motivation or sophisticated epistemic beliefs. In line with our findings, instrumental motivation was found to be positively or negatively associated with science achievement, which needs to be appropriately researched. Overall, the findings support the importance of recognizing both cultural universals and about cultural/contextual differences (Yang, 2016).

## Limitations

Some limitations should be noted. First, our findings showing the importance of intrinsic motivation and sophisticated epistemic beliefs in facilitating science learning need to be replicated across different ages as PISA focuses on 15-year old students. Second, it might also be useful to test validity using confirmatory factor analysis and explore these relationships across different regions. There are 72 regions included in PISA and we decided to focus on only four regions especially because adding more regions would make our discussion unwieldy. Societies can differ on so many dimensions (e.g., government system, colonial background, GDP per capita, income inequality, ethnicity, demographic factors). However, future studies can examine the commonality of the results to other cultural contexts. Third, our study uses a cross-sectional correlational design and we cannot make causal conclusions. Future studies can utilize longitudinal or experimental designs to establish stronger causal conclusions. Fourth, because we relied on secondary data from PISA, the current study is also limited by PISA’s sampling design and analytic framework.

## Conclusion

Our study demonstrates that, to enhance science achievement, students need to be both intrinsically motivated and possess sophisticated epistemic beliefs. This pattern is common across the selected regions with notable differences in cultural contexts. However, instrumental motivation in the present study shows a regionally specific pattern. It seems that instrumental motivation was more adaptive in Western than Eastern societies. Our study suggests both commonality and specificity and indicates that increasing students’ intrinsic motivation in science learning and helping them develop more sophisticated epistemic beliefs might be promising pathways to optimizing science achievement. This may also provide implications for science educators to motivate students’ intrinsically to learn science and incorporate pedagogical strategies that will enhance more sophisticated and deeper epistemic processes and judgment.

## Data Availability Statement

Publicly available datasets were analyzed in this study. This data can be found here: https://www.oecd.org/pisa/data/2015database/.

## Author Contributions

All authors listed have made a substantial, direct and intellectual contribution to the work, and approved it for publication.

## Conflict of Interest

The authors declare that the research was conducted in the absence of any commercial or financial relationships that could be construed as a potential conflict of interest.

## Footnotes

1 https://www.oecd.org/pisa/data/2015database/

## Appendix

**TABLE A1:** The three latent variables and their assessment items in PISA 2015.

1. Intrinsic motivation	
ST094Q01NA	I have fun when I am learning <broad science>
ST094Q02NA	I like reading about <broad science> topics.
ST094Q03NA	I am happy working on <broad science> topics.
ST094Q04NA	I enjoy acquiring new knowledge in <broad science>.
ST094Q05NA	I am interested in learning about <broad science>.
2. Instrumental motivation	
ST113Q01TA	Making an effort in my <school science> subject(s) is worth it because this will help me in the work I want to do later on
ST113Q02TA	What I learn in my <school science> subject(s) is important for me because I need this for what I want to do later on
ST113Q03TA	Studying my <school science> subject(s) is worthwhile for me because what I learn will improve my career prospects.
ST113Q04TA	Many things I learn in my <school science> subject(s) will help me to get a job.
3. Epistemic beliefs	
ST131Q01NA	A good way to know if something is true is to do an experiment.
ST131Q03NA	How much do you disagree or agree with the statements below? Ideas in <broad science> sometimes change.
ST131Q04NA	Good answers are based on evidence from many different experiments.
ST131Q06NA	It is good to try experiments more than once to make sure of your findings.
ST131Q08NA	Sometimes <broad science> scientists change their minds about what is true
ST131Q11NA	The ideas in <broad science> science books sometimes change.
